# Reidentification of Avian Embryonic Remains from the Cretaceous of Mongolia

**DOI:** 10.1371/journal.pone.0128458

**Published:** 2015-06-01

**Authors:** David J. Varricchio, Amy M. Balanoff, Mark A. Norell

**Affiliations:** 1 Department of Earth Sciences, Montana State University, Bozeman, Montana, 59717, United States of America; 2 Department of Anatomical Sciences, Stony Brook University School of Medicine, Stony Brook, NY, 11794, United States of America; 3 Division of Paleontology, American Museum of Natural History, New York, NY, 10024, United States of America; University of Pennsylvania, UNITED STATES

## Abstract

Embryonic remains within a small (4.75 by 2.23 cm) egg from the Late Cretaceous, Mongolia are here re-described. High-resolution X-ray computed tomography (HRCT) was used to digitally prepare and describe the enclosed embryonic bones. The egg, IGM (Mongolian Institute for Geology, Ulaanbaatar) 100/2010, with a three-part shell microstructure, was originally assigned to Neoceratopsia implying extensive homoplasy among eggshell characters across Dinosauria. Re-examination finds the forelimb significantly longer than the hindlimbs, proportions suggesting an avian identification. Additional, postcranial apomorphies (strut-like coracoid, cranially located humeral condyles, olecranon fossa, slender radius relative to the ulna, trochanteric crest on the femur, and ulna longer than the humerus) identify the embryo as avian. Presence of a dorsal coracoid fossa and a craniocaudally compressed distal humerus with a strongly angled distal margin support a diagnosis of IGM 100/2010 as an enantiornithine. Re-identification eliminates the implied homoplasy of this tri-laminate eggshell structure, and instead associates enantiornithine birds with eggshell microstructure composed of a mammillary, squamatic, and external zones. Posture of the embryo follows that of other theropods with fore- and hindlimbs folded parallel to the vertebral column and the elbow pointing caudally just dorsal to the knees. The size of the egg and embryo of IGM 100/2010 is similar to the two other Mongolian enantiornithine eggs. Well-ossified skeletons, as in this specimen, characterize all known enantiornithine embryos suggesting precocial hatchlings, comparing closely to late stage embryos of modern precocial birds that are both flight- and run-capable upon hatching. Extensive ossification in enantiornithine embryos may contribute to their relatively abundant representation in the fossil record. Neoceratopsian eggs remain unrecognized in the fossil record.

## Introduction

A small egg, IGM (Mongolian Institute for Geology, Ulaanbaatar) 100/2010, from the Late Cretaceous Khugenetslavkant locality, Mongolia with a fauna dominated by the necoceratopsian *Yamaceratops dorngobiensis* [1], was originally assigned to Neoceratopsia [2]. The isolated egg measures 4.75 by 2.23 cm and has a volume of 12.4 cm^3^. The egg was considered to be symmetric with sparse, randomly distributed nodes on its surface. Although the surface of the egg is heavily encrusted with sandstone externally this texture was discerned from a few “clean” areas. The microstructure of the 183–184 μm-thick eggshell consists of three structural layers with a lower layer of blocky, blade-shaped crystals forming spherulites, a thicker middle zone with larger and more numerous vesicles and a sub-horizontal crystal orientation, and a thin external zone exhibiting vertically oriented crystals. High-resolution X-ray computed tomography (HRCT) was used to digitally prepare and describe the enclosed embryonic bones, a few of which are visible at one broken terminus of the egg, from three-dimensional (3D) volumetric renderings of the skeletal elements (Balanoff et al. [2], Fig 2). The specimen was tenatively diagnosed as a neoceratopsian based on a straight quadrate shaft and the presence of a predentary. A small fourth trochanter on the midshaft region of the femur also supported this identification [2].

These interpretations represented the first associated embryo and egg for Neoceratopsia. Earlier assignments of eggs to this clade proved to be in error as demonstrated by Norell et al. [3] or, in the case of the egg form *Protoceratopsidovum*, remained controversial because taxonomic identification was based merely on the abundance of the skeletal remains in the same formation with the eggs rather than an in ovo embryo [4]. The presence of three microstructural layers in combination with a symmetric egg would have falsified the neoceratopsian identification of the *Protoceratopsidovum* egg type, which is asymmetric and composed of only two structural layers. Several research teams [5–8], however, have independently concluded that three microstructural layers of the form found in IGM 100/2010 are apomorphic for Maniraptora or a less-inclusive clade containing Aves (Note: Use of taxonomic nomenclature follows Sereno [9] and O'Connor et al. [10]). Thus, the neoceratopsian identification of the egg would suggest homoplasy among eggshell characters across Dinosauria as discussed in Balanoff et al. [2].

Given the important implications of the taxonomic assignment of this egg, we re-examined the specimen, rendered additional elements from the HRCT, and here provide further description and reevaluation of the embryo. It can now be confidently identified as that of an enantiornithine bird, thus eliminating the apparent conflict in eggshell characters among Dinosauria. Three factors contribute to the original misidentification of the embryo: 1) The orientation of the embryo was incorrectly identified. Proper orientation reverses cranial and caudal (i.e., the original forelimbs actually represent the hindlimbs, and the original hindlimbs the forelimbs). Additionally, a series of somewhat in situ axial elements become caudal dorsal and sacral vertebrae rather than caudal cervical vertebrae. Misalignment of the egg led to incorrect identification of the elements that were subsequently used to diagnose the specimen. 2) Several bones situated in the most cemented parts of the egg were not originally segmented. Review of the original scans available at the Digital Morphology website (www.digimorph.org/specimens/neoceratopsian_egg/) (S1 and S2 Movies) however, reveals that these elements include the left radius, tibiae, and metatarsals. 3) Additionally, the postcranial skeleton was underutilized in the original description, missing several characters that appear to be diagnostic for Enantiornithes. In modern birds, ossification of the embryonic skeleton begins first in the major limb bones, then the axial column, and finally the cranium [11]. The pattern in crocodilians is similar, with ossification beginning with the dermatocranium and major limb elements. Ossification then proceeds through the axial skeleton, the neuro- and splanchnocranium, the pectoral girdle, and finally the pelvic girdle [12]. Precocial hatchlings of birds possess limb elements that are recognizable as specific bones, while major portions of the cranial elements are still composed of cartilage [11]. Thus, if embryonic cranial elements are present and can be used for taxonomic assignment, major postcranial elements also should be recognizable (but see Bever and Norell [13]).

Reexamination of cranial and postcranial elements of IGM 100/2010 reveal a number of synapomorphies for successively nested clades that point towards an enantiornithine identification. Although the identifications of much of the embryonic skeleton has changed, this specimen still affords the best view of an avian embryo from the Mesozoic and allows reexamination of several hypotheses on the evolution of reproduction involving development, the precocial to superprecocial nature of hatchlings, and parental care in this important clade.

## Materials and Methods

**Fig 1:**
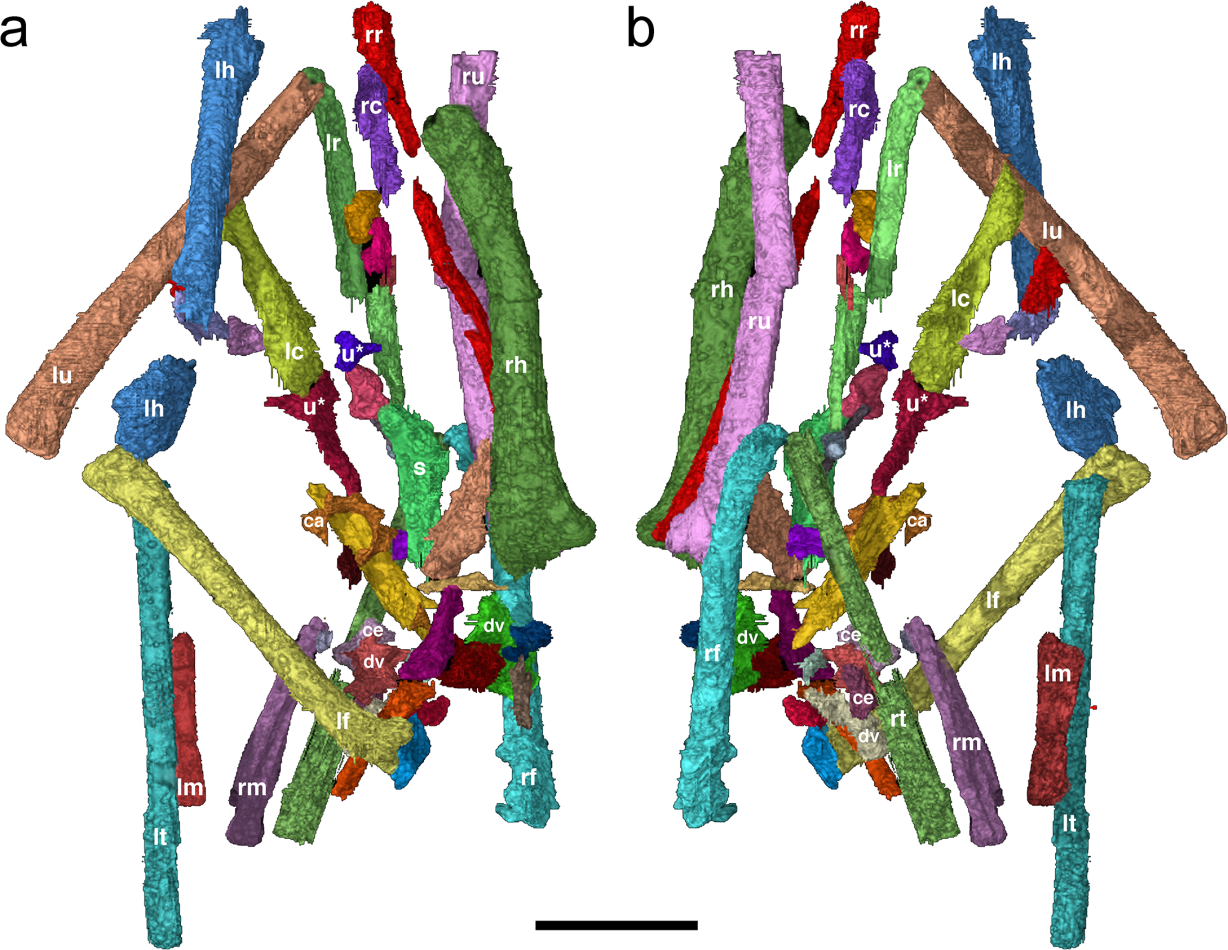
Three dimensional digital rendering of IGM 100/2010. Three dimensional digital rendering of IGM 100/2010 embryo by Balanoff et al. [2] showing the bone identification proposed here in **a,** dorsal oblique view and **b**, ventral oblique view. Long axis of embryo oriented with cranial at the top. Abbreviations: **ca**, cervical neural arch; **ce**, centrum; **dv**, dorsal vertebra; **lc**, left coracoid; **lh**, left humerus; **lm**, left tarsometatarsus; **lr**, left radius; **lt**, left tibia; **lu**, left ulna; **rc**, right coracoid; **rf**, right femur; **rm**, right tarsometatarsus; **rr**, right radius; **rt**, right tibia; **ru**, right ulna; and **s**, possibly a portion of the scapula. Note: u* marks the "predentary" (blue) and "quadrate" (red) identified by Balanoff et al. [2], but considered unidentifiable here. Scale bar equals 5 mm. Element colors match those in S3 Movie.

**Fig 2:**
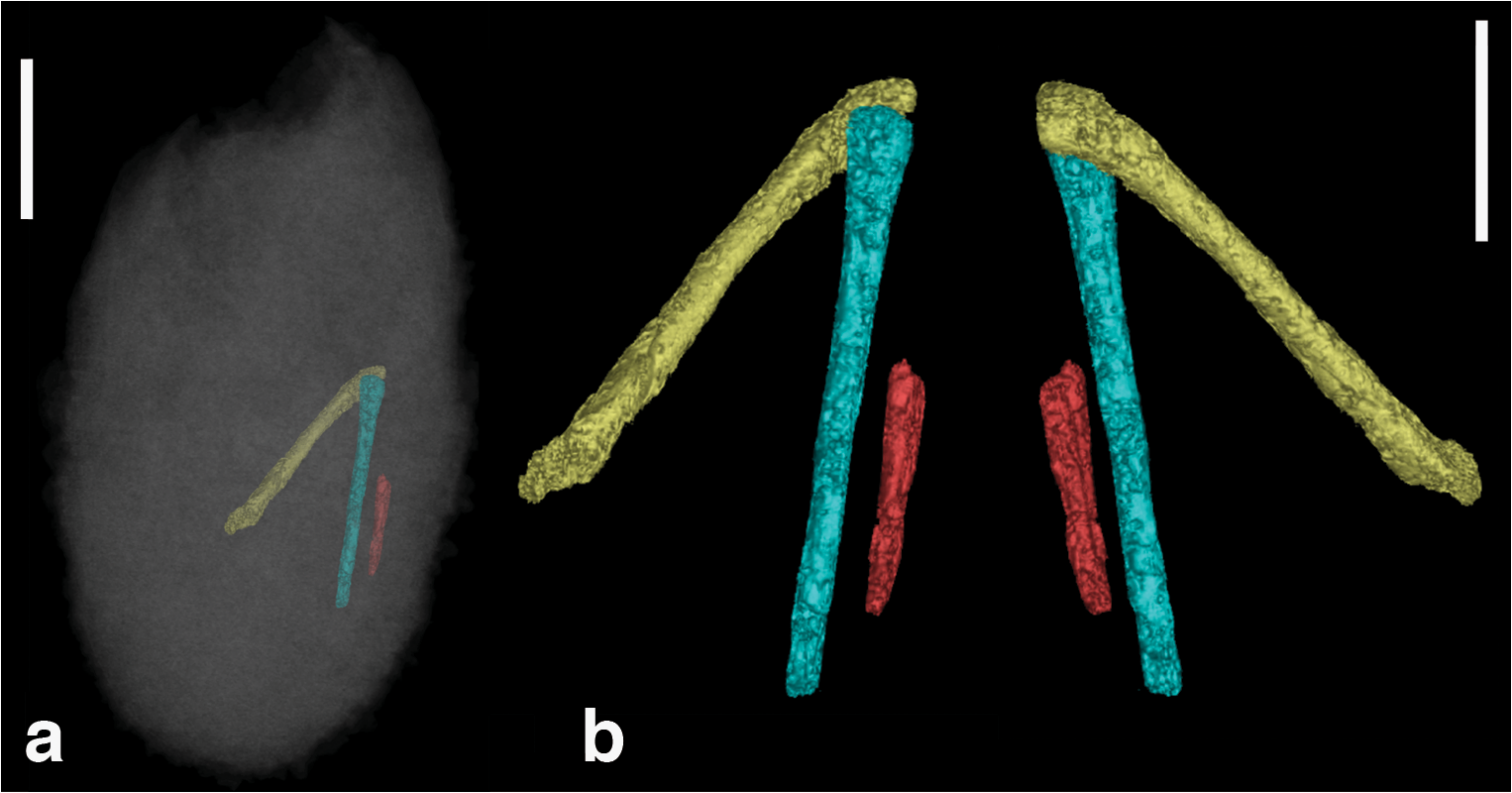
Right hindlimb of IGM 100/2010. Right hindlimb of IGM 100/2010 highlighted within the egg (**a**) and enlarged (**b**) showing the femur (yellow), tibia (blue) and metatarsus (red) in ventral and dorsal view. Scale bars equal 10 mm and 5 mm, respectively.

The specimen IGM 100/2010, belongs to the Mongolian Institute for Geology, Ulaanbaatar, but was examined on loan to the American Museum of Natural History in New York. Details on the scanning and reconstructions of this specimen are detailed in Balanoff et al. [2]. The specimen was digitally prepared with HRCT. Rendering of individual bone requires choosing and following each element throughout the series of two-dimensional slices in which it appears. Original slice data are available on request from the authors. All new skeletal identifications are presented in Fig 1, a relabeled version of the figure in Balanoff et al. Fig 2 [2]. Because many of the elements are incompletely ossified and/or lie within a complex of other bones animations of the original CT images as well as rotations of the three-dimensional (3D) renderings are available at the Digital Morphology website (www.digimorph.org/specimens/neoceratopsian_egg/). Balanoff et al. Fig 2 [2] rendered just over 40 discrete elements in 3D, but both left and right tibiae and metatarsal sets as well as the left radius, elements not presented in the original publication, are included here. The original CT slices reveal another ten or so bones within the calcareous cement that proved difficult to render volumetrically. In total, only about 50 discrete elements are visible, thus a large portion of the embryonic skeleton remains unossified, was taphonomically removed prior to burial, or lacked sufficient contrast with the matrix. Approximately 25 of the isolated bones can be identified with confidence with most representing the major elements of the limbs (Fig 1, S3 Movie). Note that element identifications used in Balanoff et al. [2] are placed in quotes, e.g. “femur”, to distinguish them from our current identifications.

## Results

In the egg, the embryo sits with the cranial end of the long axis of its torso directed toward the broken portion. As a result, the proximal ends of the humeri and distal ends of the ulnae are diagenetically truncated. Long bones are arranged close to anatomical position with proper bilateral arrangement of all four limbs. Proximal portions of both humeri and femora angle towards the midline (femoral heads separated by a space of ~4 mm), suggesting that the articulating girdle elements either are largely unossified or have been subsequently displaced. The right ulna and radius and left ulna are also visible in the 3D renderings (Fig 1, S3 Movie). The left radius is displaced with only its distal end near the distal ulna. The majority of the element has shifted away from the midline. Nevertheless, the lower leg can be traced in articulation with the distal and colorized femora (Figs 1 and 2).

The forelimbs lie folded and parallel (right) to near parallel (left) to the axial column with the elbow directed caudally. The hindlimbs are similarly oriented but with the knee directed cranially and slightly ventrally. In this arrangement, the elbows lie dorsal to the distal femora in a natural anatomical position similar to postures preserved in *Mei long* [14–15] embryonic enantiornithines [16], and in the embryos of extant birds [11] as opposed to the more unnatural posture originally posited.

A series of smaller elements mark the midline area between the contralateral sets of limbs. Identifiable elements in this region include newly identified left and right coracoids, a neural arch, three vertebrae, and a centrum (Fig 1, S3 Movie). Most of the vertebral elements lie between the proximal femora, suggesting they likely represent dorsal vertebrae. Other bones may represent portions of the manus, shoulder and pelvic girdles, and skull, but generally lack sufficient definition for precise identification. It is among this collection of midline elements, that the three cranial elements identified by Balanoff et al. [2] occur.

**Fig 3:**
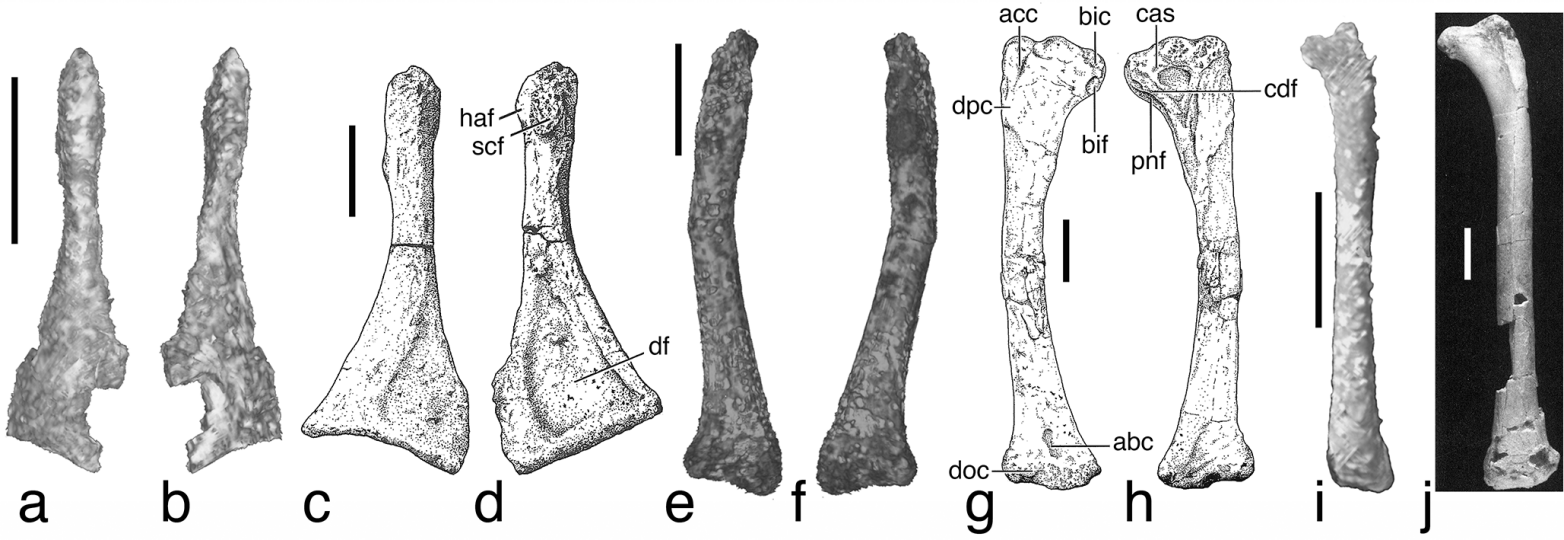
Limb element comparison of IGM 100/2010 with other Mesozoic birds. Comparison of limb elements from IGM 100/2010 to known Mesozoic bird specimens including the left coracoid of IGM 100/2010 (**a**, **b**) and the enantirnithine *Elsornis keni* (**c**, **d**) in ventral (**a**, **c**) and dorsal (**b**, **d**) view, the right humerus of IGM 100/2010 (**e**, **f**) and *Elsornis keni* (**g**, **h**) in cranial (**e**, **g**) and caudal (**f**, **h**) view, and the left femur in cranial view of IGM 100/2010 (**i**) and the ornithothoracine *Vorona berivotrensis* (**j**). Scale bars in **a**, **b**, **e**, **f**, and **i** equals 5 mm. Scale bar in **c**, **d**, **g**, **h**, and **j** equals 10 mm. *Elsornis* and *Vorona* images from respectively Chiappe et al. [36] and Forster et al. [37].

We did not find any cranial elements that could easily be identified based on morpological features or position relative to other bones. The "predentary" of Balanoff et al. [2] is a small Y-shaped element sitting medial to the proximal left coracoid (Fig 1). The bone consists of a narrow, bluntly terminating central projection and two short stout branches. The latter together present a small triangular platform when viewed on end. This platform and the similar lengths of the three projections seem inappropriate for a neoceratopsian predentary, however little is known of what this element would have looked like in early growth stages. (See Makovicky and Norell [17], fig 12; Balanoff et al. [2], Fig 3D]. The bilateral symmetry of the element does indicate a midline element. Other potential identifications could include a basisphenoid, part of a neural arch, pygostyle, or a portion of the sternum or furcula.

The element originally identified as the quadrate is an L-shaped bone in “lateral” view with a lamina extending between the two arms (Fig 1). The short, slightly arched arm sits just below the proximal end of the left coracoid and angles away from the long arm so that the angle between the two exceeds 90°. The element expands at the corner junction of the two arms in a direction perpendicular to the plane defined by these arms. The long arm terminates with a rounded end. This element bears minimal resemblance to the quadrate of *Yamaceratops* which, like those of other neoceratopsians, has a nearly straight shaft with a broad triangular pterygoid wing extending off the shaft for most of its length (Makovicky and Norell [17], fig 9). The corner expansion and blunt termination of the long arm would be consistent with an avian quadrate with a sharp, ventrally-oriented pterygoid process. However, the element also appears similar in size and form with an embryonic ischium described from stem-group bird, likely enantiornithine, from the Cretaceous of Argentina, MUCPv-284 (Museo de Geología y Paleontología, Universidad Nacional del Comahue, Neuquén, Argentina) (Schweitzer et al. [18], Fig 2).

Portions of five vertebrae are identifiable within the central collection of smaller elements. The "braincase" of Balanoff et al. [2] is somewhat ambiguous and could represent either braincase elements or a neural arch from the cervical series. This arch has a large neural canal and a low neural spine running its entire length. In dorsal view, the arch has, save for the neural spine, a near horizontal surface with a squarish outline and what appear to be pre- and postzygopophyses located at the cranial and caudal corners, respectively. Diapophyses appear to be located low on the cranial portion of the arch. The square and nearly flat dorsal aspect, low neural spine and wide neural canal are consistent with this element being a cervical arch. The remaining three vertebrae and isolated centrum lie between the proximal femora and share a similar morphology with one another. The centra are spool shaped with an approximately flat dorsal aspect. Articular faces of the centra are flat and largely semi-circular in outline, with one caudal face being somewhat more heart shaped. The neural canals are significantly narrower than that of the isolated “cervical arch”, but are nevertheless large, being similar in size to the centrum face. Two vertebrae have well-developed neural spines that make up nearly half of their entire height. The diapophyses on one vertebra project laterally from a position just dorsal to the neural canal. One isolated centrum sits in articulation with one of the complete vertebrae. These vertebrae likely represent an incomplete series from the posterior dorsals to portions of the sacrum.

Portions of both coracoids are preserved towards the cranial end of the specimen. The right coracoid consists only of the blunt proximal or omal portion that would have articulated with the scapula and furcula. More of the left coracoid is preserved but it still lacks that portion articulating with the sternum (Fig 3). The coracoids are oriented with the scapular portions projecting cranially in approximate anatomical orientation; however, the left has seemingly rotated about its long axis. The sternal portion of the coracoid flares into a thin triangular expanse that is concave on one side forming a deep coracoid fossa, and convex on the other surface. Cranially the bone narrows to a thicker shaft, then expands into a stout, asymmetric wedge of the omal end (S3 Movie). A small element at the current level of the distal humeri, might represent a portion of the scapula. The stout, blade-like fragment bears a terminal thickening as would be expected in a scapula. This fragment could also potentially represent portions of the pelvic girdle.

Due to damage to the egg, both humeri lack approximately the proximal-most 20% of their length. Nevertheless, the humerus is clearly the most massive element within the skeleton (Table 1; Fig 3). The shaft is broader mediolaterally than craniocaudally, giving it a flat, oval cross-section. The shaft bends gently in an S-shape within the mediolateral plane. Proximally, the humerus is slightly concave on its cranial aspect, and a low deltopectoral crest projects craniodorsally. The distal end of the humerus expands greatly within the mediolateral plane. In lateral view, the distal end curves cranially and bears a shallow olecranon fossa. The medial condyle of the humerus extends farther distally giving the distal end an angled profile; this may reflect the presence of flexor process. The distal condyles are located on the cranial aspect of the humerus.

**Table 1:** Element dimmensions in mm.

Element	Greatest Length	Midshaft Diameter	Distal Width
**Rt. Humerus**	14.4* (est. 17–18)	1.6	3.1
**Rt. Radius**	18.5	0.52	
**Rt. Ulna**	16.4*	1.2	
**Rt. Femur**	13.0	1.0	
**Lt. Femur**	13.4	0.92	
**Rt. Tibiotarsus**	15.6		
**Lt. Tibiotarsus**	15.5		
**Rt. Metatarsals**	8.3		
**Lt. Metatarsals**	8.8		

*Incomplete elements.

The ulna expands slightly mediolaterally at its proximal end and has a bowed shaft. The radius has a slightly mediolaterally expanded proximal end and a rounded distal end. The narrow shaft of the radius is less than half that of the ulna (Table 1). Some small elements in the area between the limbs could represent carpals or other aspects of the manus given their general location, but these lack any defining morphology that would aid in their identification.

No elements can be clearly identified as representing part of the pelvic girdle. The femur is a relatively short, narrow element, approximately 75% the length of the humerus (Table 1; Fig 3). The femoral head projects 90° relative to the shaft and sits level with the weakly developed trochanteric crest. The femoral shaft is straight with a caudally curved distal end. The equally-sized distal condyles face caudally, and the smooth cranial aspect of the distal end lacks a patellar groove. The left femur appears to bear a small foramen just proximal to mid length on the caudal aspect of the shaft, but this may represent an artifact of 3D rendering.

The remainder of the hindlimb was encased in calcareous cement, therefore, Balanoff et al. [2] did not generate 3D reconstructions of the remaining elements. We have reconstructed these elements here, but because of the poor preservational state few details can be gleaned concerning their anatomy (Fig 2). The tibia is an elongate element with a straight shaft. No signs of a fibula can be recognized in either of the otherwise articulated hindlimbs. The metatarsals are closely appressed and appear relatively straight over the central portion of their shafts.

## Discussion

Several factors allow re-identification of this embryo as a enantiornithine. Some of the elements were incorrectly identified originally because the resolution with this type of dataset can result in bones that superficially resemble other elements (e.g., "predentary" and "quadrate") or because of the mis-orientation of the specimen (e.g., “humerus” and “femur”). The major limb elements in IGM 100/2010, however, are not consistent with what one would expect in a neoceratopsian. This is particularly important given that the postcranium is better ossified than the cranium [2]. For example, the "humerus", here re-identified as the femur, lacks a deltopectoral crest, curvature of the shaft, and a mediolaterally expanded distal end, features common to neoceratopsians (Chinnery and Horner [19], fig 4; Senter [20], Fig 3). The "radius" and "ulna" appear too gracile for a neoceratopsian. Finally, a neoceratopsian femur should have a relatively straight shaft, prominent fourth trochanter, and distal condyles expanded craniocaudally (Chinnery and Horner [19], fig 4), which are absent in the element here identified as the humerus. Finally, the reversed fore- and hind limb identification results in an unusual, unnatural posture with the elbows tucked beneath the knees.

Reversing the limb identification results in anatomical identifications more consistent with that expected in an avian dinosaur (Fig 3). First, the forelimb now becomes significantly longer than the hindlimbs; humerus plus radius equals 36 mm in comparison to 29 mm for the femur plus tibia (Table 1). These limb proportions suggest an avian identification. Additionally, the postcranium provides several apomorphies that allow diagnosis of the embryo to Enantiornithes (Table 2). The strut-like coracoid and cranial placement of the humeral condyles mark the specimen as avian [10]. The presence of an olecranon fossa and a slender radius relative to the ulna further confirm this [10]. Two additional features supporting this identification include a fused, trochanteric crest on the femur and an ulna as long or longer than the humerus [10]. Neither a complete ulna nor humerus is present; however based on comparisons with other enantiornithine humeri, approximately one-fifth of the humerus is missing. The complete radius provides an approximate length for the ulna. Presence of a deep, dorsal coracoid fossa, a wedge-shaped omal portion of the coracoid with proximodistal alignment of the articular facets, and a craniocaudally compressed distal humerus with a strongly angled distal margin support a diagnosis of IGM 100/2010 as an enantiornithine [10]. The absence of prominent patellar groove on the femur, a derived feature of Ornithuromorpha [10], is also consistent with this taxonomic identification. The overall similarity of the humerus and coracoid to those of enantiornithines provides further support of this diagnosis (Walker [21], Fig 2; [22]; Fig 3).

**Table 2:** Apomorphies present in the IGM 100/2010 embryo.

**AVES** (less *Archaeopteryx*)
1. Coracoid shape strut-like.
2. Humeral distal condyles mainly located on cranial aspect.
**ORNITHOTHORACES** or perhaps **PYGOSTYLIA**
3. Ulna nearly equivalent to or longer than humerus.
4. Ulnar shaft, radial-shaft/ulnar-shaft ratio smaller than 0.70.
5. Femoral anterior trochanter forming a trochanteric crest.
**ORNITHOTHORACES**
6. Well-developed olecranon fossa on caudal face of the distal end of humerus.
**ENANTIORNITHES**
7. Broad, deep fossa on the dorsal surface of the coracoid.
8. Proximal coracoid with distinct wedge shape and proximodistal alignment of articular facets.
9. Distal end of the humerus very compressed craniocaudally.*
10. Humerus ventrodistal margin projected significantly distal to dorsodistal margin, distal margin angled strongly ventrally.

*Also found in some basal members of Ornithuromorpha. Characters from O'Connor et al. [10].

The re-diagnosis of this specimen highlights the importance of cataloguing CT data in online repositories such as Digimorph.org, providing transparency in the process of taxonomic assignment. Because of its small size and the poor resolution of the 3D renderings, the embryonic skeleton of IGM 100/2010 is a difficult specimen with which to work and obviously benefitted from further study. Additional researchers were able to identify previously unrecognized elements (e.g., metatarsus; Fig 2), which clarify the diagnosis of this specimen.

Recognition of this egg and embryo as belonging to Enantiornithes has several implications for reproduction in stem-group avians. First, it eliminates the homoplasy of the tri-laminate eggshell structure implied by a neoceratopsian identification. It instead associates enantiornithine birds with eggshell microstructure composed of a mammillary layer and squamatic and external zones. The phylogenetic and biologic significance of a third or external zone in eggshell has been a point of some debate. Presence of an external zone, a consistent feature in the eggshell of modern birds, has been considered an indication of a phylogenetic position closer to modern neognath birds than to enantiornithines [23]. Consequently, a small egg, IGM 100/1027, from the Late Cretaceous Bayn Dzak locality of Mongolia was considered more derived than Enantiornithes based on its three-layered microstructure [23]. However, eggs from the Cretaceous of Argentina, likely attributable to Enantiornithes based on embryonic remains, also exhibit a similar three-layered microstructure [18]. IGM 100/2010 concurs with Schweitzer et al. [18], and other recent papers that describe this external zone in non-avian maniraptorans [24–25] by considering the third layer to be diagnostic of a more inclusive clade within Maniraptora. Notably, Mikhailov [26–27] observes a third layer in small Cretaceous eggs from Mongolia, some of which were found with enantiornithine adults and embryos. He, however, considers this third layer to represent an artifact of diagenesis and maintains that in both IGM 100/1027 and 100/2010 it is due to recrystallization [27]. The growing number of eggs from three different continents (Asia, North and South America) and a variety of taxa displaying an external zone argue against a strictly diagenetic interpretation for this feature [28].

Surprisingly, given the rarity of both dinosaur embryos and avian skeletons, embryonic skeletons within eggs exist for several enantiornithines of the Cretaceous [16, 18, 29–30]. Among these are specimens assigned to *Gobipteryx minuta* [16] and *Gobipipus reshetovi* within *Gobioolithus minor* eggs, both from the Late Cretaceous Barun Goyot Formation of the Mongolian Gobi. Some of these eggs preserve almost complete skeletons. The IGM 100/2010 skeleton consists of most major elements of both fore- and hindlimbs, both coracoids, a possible scapula, several vertebral elements, and a scattering of smaller unidentified elements. Major missing elements include cranial bones, most of the vertebral column, ribs, furcula, sternum, manus, pelvis, and pes. The sternum ossifies late, typically after hatching in modern birds [11]. The absence of other elements (e.g., the manus, pedal phalanges, ribs, and portions of the skull) may reflect a young developmental stage. The enantiornithine embryo from Argentina (MUCPv-284) exhibits a similar suite of skeletal elements: coracoid, furcula, humerus, radius, ulna, ischium, femur and tibia [18]. The vertebral column of IGM 100/2010 remains puzzling. The presence of three vertebrae with both centra and neural arches intact suggests that ossification of the axial column was well underway. All vertebrae are displaced as are other midline elements, potentially reflecting some post-mortem disturbance. The Argentine embryo is similarly displaced.

Postures are similar among all of these various embryos, with fore- and hindlimbs folded parallel to the vertebral column and the elbow pointing caudally just dorsal to the knees [16, 29–30]. The size of the egg and embryo of IGM 100/2010 is similar to the two other Mongolian enantiornithine eggs (Table 3). The egg size falls within the range of *Gobioolithus minor*, and Mikhailov [27] implies that IGM 100/2010 belongs to this ootaxon. Nevertheless, bone lengths between these specimens are slightly different. *Gobipteryx* and *Gobipipus* have humeral and ulnar lengths of 18.7 and 21.5 mm and 13 and 15 mm, respectively, versus approximately 17.5 mm and 18.5 mm in IGM 100/2010. Although all specimens seem to be well ossified, these differences in length could still simply reflect differences in developmental stage. Alternatively, they may represent taxonomic differences in limb proportions.

**Table 3:** Element lengths in Mesozoic avian embryos.

	IVPP V14238 Liaoning	*Gobiopipus reshetovi* PIN 4492–3	*Gobiopipus reshetovi* PIN 4492–4	ZPAL MgR-I/33 Mongolia	ZPAL MgR-I/34 Mongolia	IGM 100/2010 Mongolia	MUCPv-284 Bajo de la Carpa
**REFERENCE**	[29]	[30]	[30]	[16]	[16]	this paper	[18]
**Egg Volume (cm** ^**3**^ **)**	-	10.8	10.8	-	-	12.4	17.9
**Skull**	21	16					
**Scapula**		9		11			-
**Coracoid**	6	6				9.4	
**Humerus**	12	13		14	18.7	~17.5	-
**Ulna**	13	15		16.1	21.5		-
**Radius**	12	14		15.9		18.5	-
***Ulna/Humerus***	*1*.*1*	*1*.*15*		*1*.*15*	*1*.*15*	*1.06* *	
**Metacarpal II**		7.2			11.1		
**Metacarpal III**				7.5	11.8		
**Ilium**			7				
**Pubis**			6				
**Ischium**							5
**Femur**	11		9	-		13.2	-
**Tibiotarsus**	13		13	-		15.6	-
**Metatarsal III**	9			-		8.6	-
***Tib/Femur***	*1*.*18*		*1*.*44*			*1*.*18*	
***MTIII/Femur***	*0*.*82*					*0*.*65*	
***Humerus/femur***	*1*.*09*					*1*.*33*	

All lengths in mm.

*Actually Radius/Humerus and likely a low estimate of Ulna/Humerus.

Well-ossified skeletons characterize all known enantiornithine embryos suggesting precocial hatchlings. Elzanowski [16] argued that the degree of ossification in the shoulder girdle and forelimb elements of enantiornithine embryos was well beyond what would be expected in that of most modern birds. Consequently, he argued that *Gobipteryx* was superprecocial similar to modern megapodes, which are capable of flight upon hatching. He later proposed that superprecociality is associated with male-only parental care and that this reproductive strategy is ancestral for Neornithes [31]. The *Gobipipus* embryos compare closely to late stage embryos of modern precocial birds that are both flight- and run-capable upon hatching [30]. The IGM 100/2010 enantiornithine embryo conforms to this overall pattern exhibiting well-developed ossification in both fore- and hindlimbs and to some extent in the shoulder girdle. This extensive ossification found in enantiornithine embryos may in part contribute to their relatively abundant representation in the fossil record.

The re-identification of IGM 100/2010 highlights that no eggs or eggshell directly association with neonate or adult individuals exists for the neoceratopsian clade. The absence of any egg remains stands in marked contrast to the several specimens of very small juveniles, possibly neonates for the neoceratopsians such as *Psittacosaurus*, *Bagaceratops* and *Protoceratops*, including assemblages within possible nesting traces [32–35].

## Supporting Information

S1 Movie Sagittal slice movie through the enantiornithine egg, IGM 100/2010. (MP4)

S2 Movie Horizontal slice movie through the enantiornithine egg, IGM 100/2010. (MP4)

S3 Movie Skeletal yaw movie showing the embryonic elements of IGM 100/2010 after rendering. (MP4)
